# Beyond the Genomic Storm: Evaluating Tabernanthalog as a Potential Scaffold for Silent Neuroplasticity and Broad-Spectrum Therapy

**DOI:** 10.3390/ijms27062811

**Published:** 2026-03-20

**Authors:** Ivan Anchesi, Ivana Raffaele, Maria Francesca Astorino, Maria Lui, Marco Calabrò, Giovanni Luca Cipriano

**Affiliations:** 1 IRCCS Centro Neurolesi “Bonino-Pulejo”, Via Provinciale Palermo, Contrada Casazza, 98124 Messina, Italy; ivan.anchesi@irccsme.it (I.A.); ivana.raffaele@irccsme.it (I.R.); giovanniluca.cipriano@irccsme.it (G.L.C.); 2 Department of Biomedical and Dental Sciences and Morpho Functional Imaging, University of Messina, 98125 Messina, Italy; mastorino@unime.it (M.F.A.); mcalabro@unime.it (M.C.)

**Keywords:** tabernanthalog (TBG), psychoplastogens, non-hallucinogenic psychedelics, neurorehabilitation

## Abstract

The clinical renaissance of psychedelic medicine has highlighted the therapeutic potential of rapid-acting neuroplastogens, or “psychoplastogens,” for psychiatric disorders. However, the widespread application of classical psychedelics—such as psilocybin and LSD—and the atypical dissociative ibogaine is severely limited by their hallucinogenic properties and, particularly in the case of ibogaine, life-threatening cardiotoxicity. Addressing these limitations, Tabernanthalog (TBG) has emerged as a frontrunner in the field. This non-hallucinogenic analog of ibogaine was rationally designed to eliminate interactions with the human ether-à-go-go-related gene (hERG, KCNH2) potassium channel, thereby mitigating cardiotoxic risks. While initially characterized for its anti-addictive and antidepressant-like properties, recent data from 2024–2025 have significantly expanded its therapeutic horizon. TBG demonstrates robust efficacy in preclinical models of neuropathic and visceral pain, as well as in the rescue of cognitive deficits associated with cancer-related cognitive impairment (CRCI). TBG has shown efficacy in reversing cognitive impairments induced directly by the presence of a tumor in preclinical models, rather than by chemotherapy-specific neurotoxicity. Crucially, emerging evidence suggests that TBG’s mechanism extends beyond simple 5-HT2A receptor agonism. New findings point to a multi-target profile involving the inhibition of nicotinic acetylcholine receptors (nAChRs), positive modulation of NMDA receptors, and functional crosstalk with mGlu2 receptors. Furthermore, TBG appears to induce structural neuroplasticity without the widespread induction of immediate early genes (IEGs) seen with classical hallucinogens, suggesting a decoupling of therapeutic rewiring from the subjective psychedelic experience. This review synthesizes current preclinical evidence to discuss TBG as a promising chemical scaffold for next-generation neurotherapeutics targeting the intersection of psychiatry and neurology.

## 1. Introduction

Neuropsychiatric disorders encompass a broad spectrum of conditions—including depression, anxiety, and obsessive–compulsive disorders—linked to the dysregulation of the serotonergic system. Historically, treatment has relied on monoaminergic modulators such as selective serotonin reuptake inhibitors (SSRIs); however, their clinical utility is often limited by a slow onset of action and significant non-responder rates [1]. Beyond major depressive disorder, dysregulation of serotonergic signaling contributes to a broad spectrum of neuropsychiatric conditions, including anxiety disorders, obsessive–compulsive disorder, and substance use disorders [2]. In parallel, increasing evidence indicates that maladaptive structural remodeling of cortico-limbic circuits is a common pathophysiological thread across these diagnoses, linking stress exposure, impaired cognitive control, and vulnerability to relapse [3]. Recently, the focus has shifted toward ‘psychoplastogens,’ which rapidly restore structural connectivity in atrophied circuits [4]. These compounds have shown rapid and sustained effects not only in models of addiction, but also in paradigms of depression, anxiety-like behavior, and stress-related cognitive dysfunction, positioning them as candidates for transdiagnostic interventions across neuropsychiatric disorders [5]. Among these, ibogaine has gained attention not only for its antidepressant potential but primarily for its unique ability to suppress opioid withdrawal and drug-seeking behavior. Tabernanthalog (TBG) was originally conceived as a safer, non-hallucinogenic anti-addictive scaffold to overcome the liabilities of ibogaine [6].

To circumvent these limitations, rational drug design efforts led to the synthesis of Tabernanthalog (TBG), a water-soluble, non-hallucinogenic analog of ibogaine [6]. Initial characterization demonstrated that TBG retains the therapeutic efficacy of its parent compound in models of substance use disorder (SUD) and depression, while exhibiting a superior safety profile with no detectable hERG inhibition or hallucinogenic-like behaviors (head-twitch response) in rodents [6,7].

However, the pharmacological narrative of TBG is rapidly evolving. Emerging research from 2024 and 2025 has revealed that TBG is more than a “selective” 5-HT2A agonist. Its pharmacological footprint includes interactions with nicotinic acetylcholine receptors (nAChRs) [8] and complex crosstalk with glutamate receptors (mGlu2/3 and NMDA) [9,10], opening new therapeutic avenues beyond psychiatry. Specifically, TBG has shown unexpected efficacy in treating neuropathic pain [11] and reversing cognitive impairments induced by chemotherapy [12], indications where classical psychedelics are less established.

This review provides a comprehensive analysis of the state-of-the-art literature on TBG. We critically examine its multi-target mechanism of action, contrasting its “silent” plasticity induction with the gene-expression storms triggered by classical hallucinogens [13]. Finally, we discuss the translational implications of these findings, positioning TBG as a putative transdiagnostic tool with the potential to address comorbidities at the intersection of chronic pain, cognitive decline, and neurorehabilitation.

## 2. Literature Search Strategy and Selection Criteria

To provide a highly selective narrative review, the primary literature search was conducted exclusively using the PubMed database. To maximize specificity, the single search query Tabernanthalog was utilized. This strict approach yielded exactly 10 primary results, all of which met our inclusion criteria for empirical data or mechanistic insights and are directly cited within this paper. To provide a comprehensive context, this core selection of 10 articles was subsequently enriched by manual cross-referencing and the inclusion of broader relevant literature detailing psychoplastogen mechanisms, neuroplasticity, and related pharmacological targets. Non-English publications and non-peer-reviewed preprints were excluded from the primary synthesis.

## 3. Chemical Architecture and Safety Profile

The pursuit of safer analogs like TBG is necessitated by the intricate polypharmacology of ibogaine, which exerts its effects through a wide array of molecular targets, including serotonin transporters, opioid receptor subtypes (µ, δ, κ), NMDA receptors, and sigma receptors, a profile that not only underlies its anti-addictive potential but also its significant clinical risks [14]. To address these limitations, TBG was synthesized via a simplified analog approach, effectively stripping the ibogaine skeleton of its isoquinuclidine ring while preserving the methoxy-indole core essential for 5-HT2A receptor engagement. This structural modification resulted in a water-soluble, non-hallucinogenic compound that retains psychoplastogenic properties, while also eliminating the cardiotoxic effects [6].

The primary barrier to ibogaine’s clinical approval remains its propensity to induce fatal arrhythmias via blockade of the hERG potassium channel [15]. However, comparative electrophysiological assays have shown that while ibogaine inhibits hERG currents with an IC50 of approximately 1 µM, TBG exhibits a significantly improved safety profile with an IC50 of 148 µM, showing no significant inhibition even at very high concentrations [16]. This absence of cardiotoxic liability suggests that TBG can be administered without the intensive cardiac monitoring typically required for ibogaine treatment.

**Figure 1:**
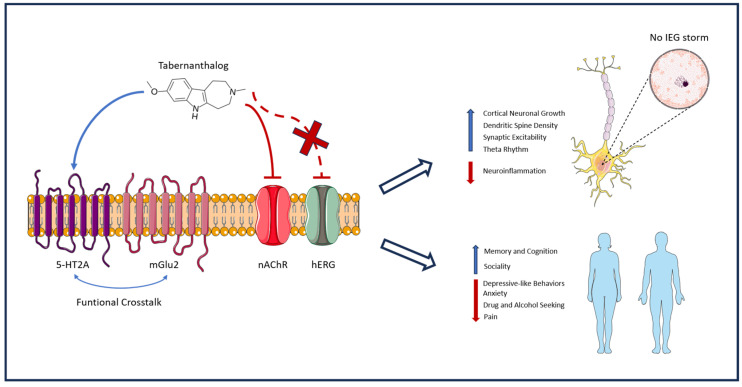
Proposed mechanism of action and therapeutic scope of tabernanthalog (TBG). TBG is depicted as a multi-target psychoplastogen that engages 5-HT2A receptors and promotes functional crosstalk with mGlu2 (indicated by curved blue arrows), while also interacting with additional receptor systems (including nAChRs and NMDA receptors). Solid red lines with flat ends denote receptor inhibition (e.g., at nAChRs), while the dashed red line with an ‘X’ signifies the lack of interaction or toxicity at the hERG channel. In contrast to classical hallucinogenic psychedelics, TBG is proposed to induce structural neuroplasticity (“silent plasticity”), supporting spinogenesis/dendritogenesis and circuit repair, without triggering a widespread immediate early gene (IEG) transcriptional surge. Vertical upward arrows signify physiological upregulation or enhancement (e.g., increased neuroplasticity, improved cognition), whereas downward red arrows indicate downregulation or reduction (e.g., decreased IEG expression, pain, and addiction behaviors). This pharmacological profile is further framed by an improved tolerability/safety rationale (minimal hERG liability and lack of hallucinogen-like behavioral proxies in rodents) and is conceptually linked to transdiagnostic benefits spanning addiction-related behaviors, neuropathic/visceral pain, and cognitive impairment. The image was created using the image bank of Servier Medical Art (available online: http://smart.servier.com/, accessed on 20 September 2025), licensed under a Creative Commons Attribution CC BY 4.0 International License (available online: https://creativecommons.org/licenses/by/4.0/).

Furthermore, TBG is distinguished by its lack of hallucinogenic potential. In rodent models, the head-twitch response (HTR) serves as the gold-standard behavioral proxy for 5-HT2A-mediated hallucinations in humans [17]. Unlike classical psychedelics such as psilocybin or 5-MeO-DMT, which induce robust HTRs [18], TBG administration across a wide dose range elicits no significant HTR in mice [6]. This lack of psychotomimetic activity is critical for therapeutic scalability, implying that TBG could potentially be prescribed for at-home use, avoiding the need for the controlled clinical settings essential for classical psychedelics. From a pharmacokinetic perspective, TBG is highly lipophilic and exhibits excellent blood–brain barrier penetrance. Following systemic administration in rodents, TBG reaches maximal brain concentrations (T*_max_*) rapidly, typically within 15 min, followed by a relatively rapid clearance (brain half-life of roughly 1–2 h) [16]. This pharmacokinetic profile is highly advantageous, as it aligns with the “hit-and-run” therapeutic paradigm of psychoplastogens: a brief pharmacological exposure is sufficient to initiate intracellular signaling cascades (such as mTOR and TrkB activation) that lead to long-lasting structural neuroplasticity and sustained behavioral changes, long after the compound has been eliminated from the system [16,19]. This complex, multi-receptor interplay orchestrates a ‘reset’ of neural circuits without the neurophysiological drama of a psychedelic trip. The convergence of these unique pharmacological mechanisms into broad therapeutic outcomes is summarized in Figure 1.

To provide a comprehensive overview of the pleiotropic effects of TBG, we synthesized the current preclinical evidence mapping specific molecular targets to their functional and behavioral outcomes. While Figure 1 illustrates the proposed mechanistic framework, Table 1 details the empirical support for the up- and downregulation of these pathways, highlighting the robust ‘silent plasticity’ profile that distinguishes TBG from classical psychedelics.

## 4. Molecular Pharmacology: A Multi-Target Orchestration

While early characterizations described TBG primarily as a 5-HT2A agonist, recent evidence from 2024–2025 depicts a far more complex pharmacological profile involving cholinergic [8] and glutamatergic modulation [10]. Regarding serotonergic signaling, TBG binds to the 5-HT2A receptor with varying affinity, yet its functional selectivity, or biased agonism, differs remarkably from that of hallucinogens like LSD [13]. While it promotes dendritic growth via 5-HT2A activation, it does so without triggering the specific downstream signaling cascade responsible for the subjective hallucinogenic experience [7].

Beyond serotonergic pathways, a pivotal 2024 study revealed that TBG also acts as an antagonist at specific nicotinic subtypes, inhibiting human α7 and α9α10 nAChRs with clinically relevant potency [8]. While the inhibition of α9α10 receptors represents a promising non-opioid target for pain and inflammation, behavioral data confirm that TBG’s primary analgesic response is driven by 5-HT2A receptor activation, as these effects are abolished by selective antagonists [11].

Finally, the therapeutic effects of TBG appear to rely on a synergistic interaction between 5-HT2A and type 2 metabotropic glutamate receptors (mGlu2). Recent findings demonstrate that the antineuropathic effects of TBG are mediated by crosstalk with mGlu2 receptors, as these effects are abolished by the selective antagonist LY341495, indicating that the molecule likely modulates the 5-HT2A-mGlu2 functional crosstalk complex [9]. The 5-HT2A-mGlu2 heteromeric complex is a critical hub for integrating serotonergic and glutamatergic signaling. Unlike classical hallucinogens that trigger unbalanced excitatory surges, TBG-mediated activation of this crosstalk appears to fine-tune glutamate release. This modulation is particularly relevant in the context of chronic pain, where the recruitment of mGlu2 signaling pathways may provide an inhibitory brake on nociceptive transmission without the dissociative side effects typical of direct NMDA antagonists. Furthermore, in the hippocampus, TBG has been shown to potentiate NMDA receptor currents and theta rhythms in CA1 pyramidal neurons. While this cellular mechanism is traditionally linked to cognitive restoration, TBG has demonstrated lower efficacy in improving spatial and recognition memory compared to other ibogalogs, such as DM506 [10].

In the broader landscape of ibogaine-inspired psychoplastogens, TBG must be contextualized alongside other recent analogs, such as Ibogainalog (IBG) and the already mentioned DM506. While all these compounds share the core objective of maintaining psychoplastogenic efficacy without hallucinogenic or cardiotoxic liabilities, they exhibit distinct pharmacological nuances. For instance, recent structural and electrophysiological analyses by Tae et al. (2024) revealed that while both TBG and IBG inhibit α7 and α9α10 nAChRs, they do so via different mechanisms and binding modes [8]. Furthermore, behavioral differentiation is emerging among these scaffolds: while TBG is highly effective in models of addiction and pain, Chagraoui et al. (2025) demonstrated that IBG and DM506 are significantly more efficacious than TBG in enhancing hippocampal-dependent spatial and recognition memory [10]. This divergence suggests that subtle structural variations among ibogalogs fine-tune their functional selectivity at 5-HT2A and NMDA receptor complexes, offering the potential to tailor specific analogs for distinct neuropsychiatric or neurological indications [8,10].

## 5. Mechanisms of Neuroplasticity: The “Silent” Rewiring

The defining feature of psychoplastogens is their ability to rapidly induce structural neuroplasticity, a capability TBG shares but executes with a unique transcriptional signature that differentiates it from classical psychedelics [6]. Functionally, TBG administration rapidly promotes spinogenesis and dendritogenesis in key cortical areas [16]. For instance, in mice subjected to unpredictable mild stress, a single dose of TBG was sufficient to reverse stress-induced spine elimination in the medial prefrontal cortex (mPFC) and restore antidepressant-like behaviors [16]. Crucially, this remodeling is now recognized as ‘silent plasticity,’ as TBG promotes spinogenesis and restores neuronal communication without triggering the expression of immediate early genes (IEGs) such as c-Fos or Arc, a key mechanistic departure from classic hallucinogenic psychoplastogens [7,13]. By facilitating this ‘gentle’ yet effective rewiring of dysfunctional networks, TBG promotes a long-term stabilization essential for neurological neurorehabilitation. This structural rescue correlates directly with the compound’s rapid antidepressant and anxiolytic behavioral effects. At the intracellular level, TBG-induced structural remodeling appears to converge on canonical neuroplasticity pathways. Non-hallucinogenic ibogalogs, including TBG, have been shown to activate signaling cascades involving TrkB and downstream mTOR, which regulate dendritic growth, synaptic protein synthesis, and spine stabilization. These pathways offer attractive pharmacological targets in their own right and may underpin the durable behavioral effects observed after brief TBG exposure [19].

Importantly, the absence of a brain-wide IEG “genomic storm” does not preclude more selective transcriptional programs downstream of 5-HT2A–glutamate crosstalk. Activation of 5-HT2A receptors in cortico-limbic networks can engage NMDA-dependent calcium influx and intracellular cascades that converge on CREB and BDNF signaling, which are core mediators of long-term synaptic consolidation and circuit remodeling [5]. In this framework, TBG may favor a spatially and temporally restricted recruitment of CREB/BDNF pathways, supporting durable neuroplasticity without the broad, transient IEG surge typically induced by classical hallucinogens [13].

However, TBG presents a significant deviation from established models through what is termed the “transcriptional paradox.” While classical psychedelics like psilocybin and LSD induce a massive, brain-wide upregulation of Immediate Early Genes (IEGs) such as c-Fos, Arc, and Egr1/2—a “genomic storm” temporally linked to the intense sensory experience of a trip—TBG avoids this response [20]. Recent evidence suggests that non-hallucinogenic analogs like TBG promote structural plasticity through a “silent” pathway. This genomic dissociation implies that the therapeutic remodeling of neural circuits (spinogenesis) can be decoupled from the rapid transcriptional bursts typically associated with the psychedelic experience. In a groundbreaking 2025 study, Aarrestad et al. demonstrated that TBG drives potent neuroplasticity without this acute, widespread IEG activation [13]. This finding challenges the central dogma of psychedelic therapy, suggesting that the molecular machinery required for growing new synapses can be uncoupled from the neuronal burst firing associated with the hallucinogenic state. This phenomenon of “silent” plasticity represents the ideal profile for a take-home neurotherapeutic, as it allows for synaptic repair without the need for the clinical supervision required during a psychedelic experience. Future work should therefore dissect how 5-HT2A–mGlu2–NMDA signaling under TBG reorganizes chromatin and transcriptional hubs, and whether a targeted engagement of CREB- and BDNF-dependent pathways can fully account for the dissociation between synaptogenesis and immediate early gene activation observed in vivo [3].

## 6. Therapeutic Spectrum: From Psychiatry to Neurology

While initially hailed as an “anti-addiction” molecule, the therapeutic scope of TBG has widened dramatically. The pharmacological profile of TBG suggests its role as a versatile agent for neurorehabilitation in conditions characterized by pathological synaptic pruning or circuit maladaptation. Recent preclinical data from 2024–2025 suggest a transdiagnostic efficacy that spans from substance use disorders to neurological conditions involving chronic pain and cognitive decline. In the realm of substance use disorders (SUD), TBG has demonstrated robust efficacy in reducing heroin seeking and alcohol drinking. Crucially, a 2023 study by Heinsbroek et al. utilized a highly translational rat model involving a two-bottle choice alcohol binge protocol combined with intravenous heroin self-administration. In this polydrug paradigm, a single dose of TBG significantly reduced the motivation to seek both heroin and alcohol during relapse testing, addressing the complex clinical reality of polysubstance abuse [6]. Unlike substitution therapies such as methadone that rely on maintaining receptor occupancy, TBG appears to “reset” maladaptive reward circuitry via structural plasticity, producing persistent effects that often outlast the drug’s short half-life [6].

Beyond addiction, the treatment of neuropathic and visceral pain represents a paradigm-shifting frontier for this molecule. In key in vivo experiments, Arias et al. (2024) assessed TBG in diverse pain modalities, demonstrating that it significantly alleviates visceral hypersensitivity in mice subjected to Dextran Sulfate Sodium (DSS)-induced colitis, as well as mitigating mechanical allodynia in the Chronic Constriction Injury (CCI) model of neuropathic pain [11]. This is a particularly critical finding given that Oxaliplatin-induced peripheral neuropathy (OIPN) results in peripheral neurotoxicity and chronic pain, remaining largely intractable with current pharmacotherapy. The study further elucidated that the anti-allodynic effect of TBG is likely mediated by the 5-HT2A receptor, as the administration of the selective antagonist ketanserin significantly abolished the analgesic response [9,11]. The efficacy is further enhanced by mGlu2 receptor activation, indicating a synergistic functional crosstalk between 5-HT2A and mGlu2 receptors that drives analgesia [9]. This highlights a sophisticated crosstalk between the serotonergic and glutamatergic systems in the processing of chronic pain [9]. Importantly, this analgesic effect is not blocked by the opioid antagonist naloxone but is abolished by mGlu2 receptor blockade [9]. This confirms that TBG combats pain via the 5-HT2A-mGlu2 functional crosstalk complex rather than the mu-opioid receptor, offering a promising alternative for chronic pain management without the risks of respiratory depression associated with traditional opioids [11].

Finally, TBG shows promise in addressing Cancer-Related Cognitive Impairment (CRCI). Arinaga et al. (2025) reported that TBG restores object recognition memory in tumor-bearing mice, specifically reversing CRCI induced by the 3LL model, rather than chemotherapy-induced neurotoxicity [12]. Further supporting this cognitive angle, Chagraoui et al. (2025) found that TBG enhances NMDA receptor function and increases theta power in hippocampal CA1 pyramidal neurons [10]. However, while these cellular mechanisms are linked to memory consolidation, behavioral evidence from the Barnes maze and Novel Object Recognition Test (NORT) indicates that TBG exhibits significantly lower efficacy in improving spatial and recognition memory compared to other ibogalogs, such as DM506 and IBG [10]. Furthermore, a critical limitation persists in these recent evaluations [10,12]: despite the clinical necessity for diverse data, researchers continue to rely exclusively on male rodent models, leaving a significant gap in the understanding of TBG’s effects across sexes.

## 7. Translational Perspectives

Despite the compelling rodent data, the “valley of death” between preclinical success and clinical approval remains steep. The translational viability of this pharmacological class is exemplified by Zalsupindole (DLX-001), a non-hallucinogenic isotryptamine psychoplastogen currently in clinical development [21]. In a Phase I study involving 106 healthy volunteers, Zalsupindole demonstrated a favorable safety profile with no reported hallucinogenic, psychotomimetic, or dissociative effects across a broad oral dose range of 2–360 mg [22]. Notably, quantitative EEG biomarkers confirmed target engagement and CNS activity consistent with the induction of neuroplasticity, providing human proof-of-concept for the “silent plasticity” hypothesis originally observed in TBG. Furthermore, preliminary Phase 1b data in patients with Major Depressive Disorder (MDD) revealed rapid and sustained therapeutic effects, with a 12-point reduction in MADRS scores observed as early as day 8 [22]. These clinical findings suggest that the structural remodeling of neural circuits can be successfully decoupled from the subjective psychedelic experience in humans. Consequently, a key challenge for future clinical trials will be to evaluate how TBG-induced plasticity can be synergistically combined with standard neurorehabilitation protocols to maximize functional outcomes.

In the broader landscape of non-hallucinogenic ibogaine analogs, TBG is frequently compared to 18-methoxycoronaridine (18-MC). However, TBG offers distinct advantages over this older compound. Most notably, TBG offers superior scalability; it can be synthesized in a single step from commercially available materials, whereas 18-MC requires a complex 13-step synthesis. Furthermore, while 18-MC is safer than ibogaine, TBG exhibits an even cleaner hERG profile and a better characterized link to rapid dendritogenesis.

Nevertheless, significant limitations in current research persist, highlighting the translational gap between rodent models and human clinical application. First, while preclinical efficacy is robust, rodent models inherently fail to fully recapitulate the complex, multifaceted nature of human neuropsychiatric and chronic pain disorders. Differences in species-specific pharmacokinetics, drug metabolism, and cortico-limbic connectivity necessitate cautious interpretation of effective dosages and long-term behavioral outcomes. Regarding sex differences, it is noteworthy that foundational studies on TBG (Cameron et al., 2021; Aarrestad et al., 2025), as well as recent evaluations (Arinaga et al., 2025), proactively included both male and female subjects, reporting no significant sex-dependent variations in structural plasticity induction [12,13,16]. However, while TBG research performs better than the historical male-biased baseline of general preclinical psychopharmacology, systematic evaluation across all complex behavioral paradigms (such as distinct modalities of chronic pain or addiction) remains essential. This is particularly crucial given the pronounced sexual dimorphism observed in human depression and chronic pain conditions (e.g., neuro-immune signaling pathways and hormonal regulation of 5-HT2A receptor density). Finally, comprehensive data on the consequences of chronic or repeated TBG administration are still lacking; confirming its long-term cardiovascular safety, particularly regarding 5-HT2B agonism-related valvulopathy, remains an essential milestone before advancing to large-scale human trials [23].

## 8. Conclusions

Tabernanthalog represents a significant development in neuropsychopharmacology. It is not merely a “safer ibogaine” but serves as a mechanistic model of a new class of precision psychoplastogens. By structurally exorcizing the cardiotoxicity and hallucinogenic liability of its parent compound, TBG has transformed a traditional plant-derived psychedelic into a scalable clinical candidate. The evidence reviewed here delineates a molecule with a unique multi-modal mechanism: it recruits neuroplasticity-related signaling (mTOR, TrkB) without the genomic storm of immediate early genes and modulates pain processing via novel cholinergic and glutamatergic crosstalk. If clinical trials confirm the translational validity of these findings, TBG-derived compounds could provide therapeutic options for conditions associated with synaptic dysconnectivity, including addiction, depression, and chronic pain. Ultimately, Tabernanthalog stands as a valuable tool for a new generation of psychoplastogens aimed at investigating the restoration of neural health in neurorehabilitation.

## Figures and Tables

**Table 1:** Mechanistic and functional landscape of Tabernanthalog (TBG). The table summarizes the directional modulation ↑ upregulation/activation; ↓ downregulation/antagonism) of key biological targets and the resulting physiological or behavioral phenotypes as reported in the recent literature (2021–2025).

Target/Process	Modulation	Functional Outcome	Study Type/Experimental Model	References
Neuroplasticity				
Cortical Neuronal Growth	↑	Increased dendritic complexity and synaptogenesis	In vitro (rat embryonic cortical cultures); In vivo (mice)	[7,16]
Dendritic Spine Density	↑	Restoration of connectivity in stressed mPFC	In vivo (mouse Unpredictable Mild Stress model)	[7,16]
Synaptic Excitability	↑	Potentiation of NMDA receptor currents in CA1	Ex vivo (rat hippocampal slices)	[10]
Immediate Early Genes (IEGs)	↓	“Silent plasticity” (lack of c-Fos, Arc, Egr1/2)	In vivo (mouse transcriptomics)	[13]
Pathophysiology				
nAChR Activity	↓	Antagonism of α7 and α9α10 subtypes	In vitro (Xenopus oocytes expressing human/rat nAChRs)	[8]
hERG Channel Inhibition	↓	Minimal cardiotoxic risk (IC50 = 148 µM)	In vitro (electrophysiology)	[16]
Neuroinflammation	↓	Reduced visceral and neuropathic hypersensitivity	In vivo (mouse CCI and DSS-induced pain models)	[11]
Behavioral Domains				
Memory and Cognition	↑	Rescue of CRCI (Rescue of disease-related deficits)	In vivo (mouse 3LL tumor-bearing model)	[12]
Depressive-like Behaviors	↓	Rapid and sustained antidepressant effects	In vivo (mouse Forced Swim Test & Tail Suspension)	[7,16]
Drug and Alcohol Seeking	↓	Reduced motivation in polydrug use models	In vivo (rat two-bottle choice/intravenous self-administration)	[6]
Chronic Pain	↓	Mitigation of mechanical and visceral allodynia	In vivo (mouse CCI and DSS-induced pain models)	[9,11]

## Data Availability

No new data were created or analyzed in this study. Data sharing is not applicable to this article.

## Abbreviations

The following abbreviations are used in this manuscript:

18-MC	18-Methoxycoronaridine
5-HT2A	Serotonin 2A receptor
5-MeO-DMT	5-Methoxy-N,N-dimethyltryptamine
Arc	Activity-regulated cytoskeleton-associated protein
CA1	Cornu ammonis 1 (hippocampal subfield)
DOI	2,5-Dimethoxy-4-iodoamphetamine
Egr1/2	Early growth response proteins 1/2
hERG	Human ether-à-go-go–related gene (KCNH2) potassium channel
HTR	Head-twitch response
IBG	Ibogaine
IC50	Half-maximal inhibitory concentration
IEG(s)	Immediate early gene(s)
LSD	Lysergic acid diethylamide
mGlu2	Metabotropic glutamate receptor 2
mGlu2/3	Metabotropic glutamate receptors 2/3
mPFC	Medial prefrontal cortex
mTOR	Mechanistic target of rapamycin
nAChR(s)	Nicotinic acetylcholine receptor(s)
NMDA	N-Methyl-D-aspartate (receptor)
NORT	Novel Object Recognition Test
OIPN	Oxaliplatin-induced peripheral neuropathy
SSRI(s)	Selective serotonin reuptake inhibitor(s)
SUD	Substance use disorder
TBG	Tabernanthalog
TrkB	Tropomyosin receptor kinase B
